# Significance of Endocan Expression in Various Types of Epithelial Ovarian Tumors

**DOI:** 10.30699/IJP.2022.540192.2740

**Published:** 2022-03-08

**Authors:** Maryam Entezarian, Fereshteh Ameli, Noraidah Masir, Tan Geok Chin

**Affiliations:** 1 Department of Pathology, Faculty of Medicine, University Kebangsaan Malaysia, Kuala Lumpur, Malaysia; 2 Department of Pathology, Cancer Institute, Imam Khomeini Hospital Complex, Tehran University of Medical Science, Iran; 3 Department of Pathology, Faculty of Medicine, University Kebangsaan Malaysia, Kuala Lumpur, Malaysia

**Keywords:** Endocan, Endothelial cell-specific molecule-1, Immunohistochemistry, Ovarian, Neoplasm, Tumor

## Abstract

**Background & Objective:**

Ovarian cancer is associated with the highest mortality rate among gynecologic malignancies. Despite new therapeutic strategies, ovarian cancer still has a high risk of metastasis and mortality. Endocan is a newly identified endothelial cell activation marker, which is responsible for angiogenesis, tumor invasion, and aggressive behavior of tumors. The aim of this study was to assess Endocan expression in different types of ovarian tumors and to identify its relationship with clinicopathologic characteristics of ovarian tumors.

**Methods:**

This cross-sectional study was conducted on 183 tissue samples, including benign, borderline, and malignant ovarian tumors collected from the University Kebangsaan Malaysia Medical Center archive of Pathology during 2005-2015. Mouse monoclonal anti-human Endocan/ESM-1 Clone MEP08 was used at a dilution of 1:400 for immunohistochemical (IHC) staining. All the information was collected by a checklist, and the association between clinicopathological features and high or low levels of Endocan -MVD was evaluated using Pearson chi-square, Fischer's exact, or Monte Carlo tests.

**Results:**

The prevalence of Endocan positivity was significantly higher in malignant compared to borderline and benign ovarian tumors (*P*<0.001). There was also a significant association between type of tumor and Endocan status in malignant ovarian tumors (*P*=0.02), indicating that Endocan positivity was more likely in serous malignant ovarian tumors compared to other ovarian tumor types. However, the tumor stage was not significantly associated with Endocan status (*P*=0.31).

**Conclusion:**

This study showed that Endocan positivity may show the highest prevalence among malignant tumors suggesting that high Endocan expression would be negatively associated with ovarian tumor behavior.

## Introduction

Epithelial ovarian cancer (EOC) has the highest mortality rate in gynecologic cancers. A high mortality rate due to EOC might be due to the difficulty in diagnosis, which results in late diagnosis and a high frequency of relapse and drug resistance in this cancer (1). Therefore, the overall survival of patients with ovarian cancer is poor (2). 

Determinants of overall survival may include cancer stage, histological type, grade, time of diagnosis, cancer management, age, presence of other comorbidities, and type of hospital (3-5). Therefore, it is crucial to consider prognostic factors in evaluating and treating patients with ovarian cancer. Despite the critical role of prognostic factors in ovarian cancer evaluation and treatment, many currently proposed prognostic factors have not yet been accepted in clinical practice (6). 

Treatment options in EOCs include chemotherapy and surgery. Although recent advances in chemotherapy and surgical management of advanced ovarian cancer have slightly improved the outcomes of EOCs, still the majority of EOCs patients die due to drug resistance (7). The treatment obstacles in EOCs have urged scientists to focus on using molecularly targeted therapies to manage EOCs (7). 

Endocan, also known as endothelial cell-specific molecule-1 (ESM1), is a soluble dermatan sulfate (DS) proteoglycan that is secreted from different cell lines, including human vascular endothelial cells, and can be detected in the bloodstream. Endocan can affect cancer initiation and progression by participating in molecular interactions in various biological procedures. Endocan has an important role in cell proliferation, adhesion, migration, and invasion, as well as regulating cancer cell survival suppressing apoptosis via the NF-κB signaling pathway (7, 8).

Endocan overexpression at either messenger ribonucleic acid (mRNA) or protein level have been observed in pituitary adenoma (9, 10), gastric cancer (11), bladder cancer (12), non-small cell lung cancer (13), colorectal cancer (14), glioblastoma (15), renal cell carcinoma (16), hepatocellular carcinoma (17), squam-ous cell carcinoma of head and neck (18, 19), pancreatic neuroendocrine tumor (20) and ovarian cancer (21). It is generally accepted that Endocan overexpression is associated with aggressive progression and poor outcomes of tumors (22). 

The prognostic value of tissue and blood angiog-enesis-associated endothelial biomarkers have been evaluated in cancer patients. For instance, the MVD on tissue sections and blood cytokine levels of vascular endothelial growth factor VEGF have been previously evaluated. MVD is mostly evaluated using antibodies against pan-endothelial cells, such as the anti-CD31, -CD34, or - von Willebrand factor antibodies. CD34 is a pan-endothelial marker of microvascular endothelial cells that is not expressed by the endothelial cells of large vessels and can be used to label newly formed and normal vessels within tumor tissues. Thus, it is valuable to find markers that produce a specific reaction only with the endothelium of angiogenic tissue not the endo-thelium of most normal tissues. Based on the findings of the study by Manal* et al.* (2013), Endocan expression in ovarian cancer tissues was associated with other prognostic factors. To the best of our knowledge, this study was the only research that evaluated the expression of Endocan on ovarian tumors based on immune-histochemistry (18). 

However, the clinical significance of Endocan in the diagnosis of several cancers is debated. 

Therefore, we conducted this study to investigate the expression of Endocan in various types of epithelial ovarian tumors and to assess the relationship between Endocan expression and clinicopathological variables, including age, histological type, and stage of the tumor, in epithelial ovarian tumors.

## Material and Methods

**Study Population**

This retrospective, cross-sectional and observa-tional study was performed on ovarian neoplasms retrieved from the medical records of the University Kebangsaan Malaysia Medical Center (UKMMC) between January 2005 and December 2015 based on a universal sampling method.

This study was approved by the Ethics Committee of the UKMMC (Student Project No: p49845). All epithelial ovarian neoplasm cases, including benign, borderline, and malignant tumors, were included in this study. However, metastatic carcinoma and cases with inappropriate or unavailable tissue blocks or clinic-pathological data were excluded from this study.

Histopathological slides of the corresponding subjects were reviewed by two pathologists to confirm the diagnosis and histopathological type of tumors. Representative areas from 183 formalin-fixed para-ffin-embedded tissue blocks were carefully selected based on H&E stained sections. Tissue microarray (TMA) was constructed by extracting 1.0 mm diameter cores of tissue from marked areas using Alpheleys TMA Booster (Plaiser, France) tissue core extractors and re-embedding these cores into recipient paraffin blocks. Control tissue from normal ovarian tissues was included in the TMA blocks. The information rega-rding the patients' age, tumor histological type, and stage were obtained from corresponding histopath-ology reports via the Laboratory Information System and/or the surgical department records. Patient data remained anonymous, and each patient was coded accordingly. 

**Immunohistochemical Staining**

Mouse monoclonal anti-human Endocan/ESM-1 Clone MEP08 (Cat. No.: LIA-0901, Lunginnov France) was used at a dilution of 1:400 for immune-histochemical (IHC) staining. Colon carcinoma was used as control tissue. Briefly, the sections were dep-araffinized, rehydrated, and subjected to heat antigen retrieval technique. Immunostaining was performed according to the standard protocols provided by the manufacturer.

**Detection and Scoring**

Endocan expression was interpreted based on intratumoral microvessel density (MVD) with a light microscope using the method described by Weidner (22). A positive reaction was defined as brown labeling of endothelial cells or endothelial cell clusters. The immunostained sections were first viewed at low po-wer (x40 magnification). Three tumor areas with the highest density of distinctly highlighted microvessels (hot spot) were selected for quantitation of angiog-enesis. All brown stained endothelial cells or endothe-lial cell clusters that were clearly separated from connective tissue elements were considered as microv-essels. The number of microvessels was counted at x200 magnification. Endocan-MVD value was determ-ined by calculating the mean of the total number of microvessels in the three hot spot areas. 

In this study, MVD values for all cases were sum-med up, and a mean value was calculated. Cases with values below the mean were considered as low Endoc-an-MVD (negative), and cases with values equal to or above the mean were considered as high Endocan –MVD (positive). Two pathologists, who were blinded about the clinicopathologic parameters and outcomes of the patients, performed the scoring. All the inform-ation regarding demographic, histopathological, and IHC findings were collected by a checklist and classified in an Excel worksheet.

The Chi-square, Fisher's exact, or Monte Carlo tests were conducted to evaluate the correlation between high or low levels of Endocan -MVD and clinical and histopathological features of the patients, including age, race, tumor size, tumor grade, and tumor stage. P-values less than 0.05 were considered statistically significant**.**

## Results

Endocan was positive (moderate to strong intensity) in 93 subjects (51%) and negative in 91 subjects (49%). 

Amongst 72 patients with benign tumors, 22 cases (30%), including 12 serous and 10 mucinous tumors, were Endocan positive. The mean age of Endocan-positive patients was 45±6 years old. There was no significant difference between age (*P*=0.97), ethnicity (*P*=0.31), and type of ovarian tumor (*P*=0.65) between Endocan positive and negative patients in benign tumors. 

Amongst 33 patients with borderline tumors, 13 patients (39%) were Endocan positive. The mean age of Endocan-positive patients was 40±15 years old. There was no significant difference in age (*P*=0.13) and ethnicity (*P*=0.47) between Endocan positive and negative patients in borderline tumors. However, there was a significant association between type of tumor and Endocan status in borderline tumors (*P*=0.01). This indicates that mucinous borderline tumors were more likely to be Endocan positive.

Amongst 78 patients with malignant tumors, 58 (74%) were Endocan positive. The mean age of Endocan-positive patients was 51±14 years old. There was no significant difference in age (*P*=0.62) and ethnicity (*P*=0.23) between Endocan positive and negative patients in malignant tumors. There was a significant association between type of tumor and Endocan status in malignant ovarian tumors (*P*=0.02). This indicates that Endocan positivity was more likely to be found in serous malignant ovarian tumors. 

**Fig. 1:**
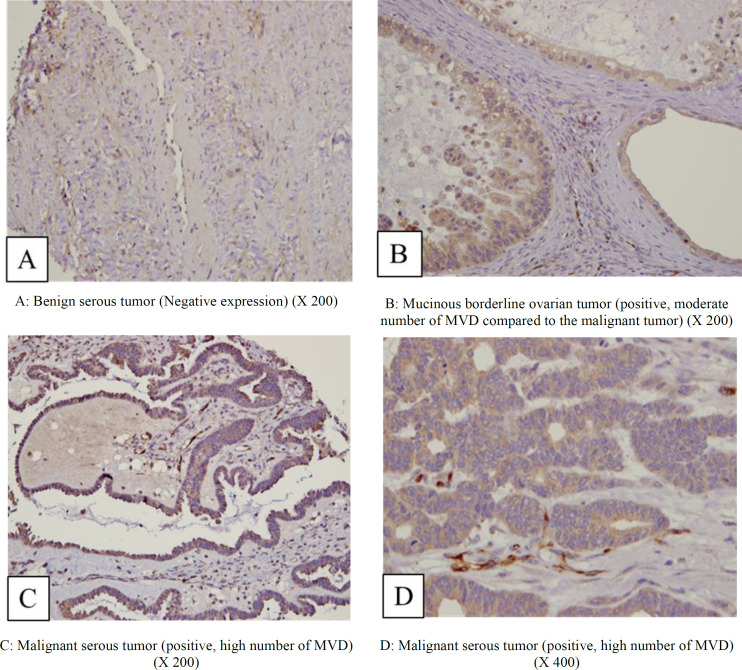
Endocan expression in vascular channels demonstrated by immunohistochemical staining (X200-X400)

Stage IV tumor was found in 18 (31%) Endocan-positive malignant patients. There was no significant association between the tumor stage and Endocan status (*P*=0.31). There was a significant difference between borderline and malignant tumors in terms of Endocan positivity (*P*<0.001). This finding indicates that the prevalence of Endocan positivity was significantly higher in malignant tumors compared with borderline and benign ovarian tumors. The results are summarized in Table 1 and Figure 1.

**Table 1:** Endocan expression in various types of ovarian tumors in relation to the clinicopathological parameters

Tumor category	Parameters/markers	High Endocan-MVD )Positive),No	Low Endocan -MVD (Negative),No	P-value
Benign Race	Malay	16	34	***P*** **=0.31**
	Chinese	3	13	
	Indian	0	2	
	Others	3	1	
Borderline Race	Malay	10	12	***P*** **=0.47**
	Chinese	3	7	
	Indian	0	0	
	Others	0	2	
Malignant Race	Malay	40	15	***P*** **=0.23**
	Chinese	13	3	
	Indian	3	1	
	Others	1	1	
Benign Histologic Type	Serous	12	31	***P*** **=0.65**
	Mucinous	10	19	
Borderline Histologic Type	Serous	4	0	***P*** **=0.01**
	Mucinous	9	19	
	Seromucinous	0	1	
	Clear cell	0	1	
Malignant Histologic Type	Serous ca	35	10	***P*** **=0.02**
	Mucinous ca	7	7	
	Endometrioid ca	13	1	
	Clear cell ca	3	2	
Malignant Tumor Stage	Stage I	30	9	***P*** **=0.31**
	Stage II	3	1	
	Stage III	7	2	
**Stage IV**	**18**	**8**

## Discussion

Endocan was first described by Lassalle* et al.* in 1996 after cloning from human umbilical vein endothelial cell complementary DNA library (23). Angiogenesis is a key event in many types of cancers. This process increases tumor blood supply so that an emerging tumor transforms from localized to the aggressive stage (24). In addition, Endocan could be considered a tumor marker and a possible new target for cancer therapy (25). While some published studies considered Endocan as a marker for angiogenesis, others considered that as a prognostic factor (19).

Furthermore, Endocan has been proposed as a predictor of cancer recurrence in patients with pancreatic neuroendocrine tumors (19). A study on colon cancer indicated that Endocan expression correlated with tumor size, depth of invasion, lymph node, distant metastases, and tumor stage (21). Irani* et al.* also showed an increased expression level of Endocan in the tumor, and endothelial cells of oral squamous cell carcinoma were significantly correlated with tumor cell differentiation (26). Endocan is therefore a unique circulating proteoglycan that appears today as a molecule of versatile interest in the study of tumor progression (19).

In a meta-analysis by Xing Huang* et al.* in 2016, the findings of 15 studies on various types of cancers were combined (overall 1,464 patients). The meta-analysis suggested elevated Endocan as a predictor of poor overall survival in patients with cancer (25).

The results of studies that evaluated the association between MVD of other biomarkers, including CD34, CD31, and CD105, and epithelial ovarian cancer prognosis, were controversial (19, 27). For instance, CD105, a marker for proliferating endothelial cells and neoangiogenesis, was found to be an independent prognostic factor for tumor progression and survival in women with advanced EOCs after adjusting for prognostic clinical covariates; however, such an association was not observed between CD31, a pan-endothelial marker, and tumor progression and survival (28).

To the best of our knowledge, only one study has been published on immunohistochemically Endocan expression in ovarian tumors , which introduced Endocan-MVD as an independent prognostic marker for overall survival of epithelial ovarian cancer (*P*<0.01). Overall survival of patients in this study was inversely associated with Endocan-MVD (*P*<0.01). They also found that Endocan was only expressed in ovarian cancer tissue endothelium in all subjects and that no Endocan expression was observed in the endothelium of normal ovarian tissues (19). 

Our study revealed that Endocan was positive in 29%, 43%, and 71% of benign, borderline, and malignant ovarian tumors, respectively. This finding showed an upward trend indicating that Endocan expression level was elevated by increased tumor aggressiveness. These results were relatively in accordance with previous studies on ovarian cancer (22), oral cancer (22), and non-small-cell lung cancer tumors (19). Furthermore, strong Endocan expression in high-grade serous carcinoma of the ovary, which has the worst prognosis among EOCs, could be supportive of this association and another proof for the hypothesis that Endocan can be a predictor for tumor prognosis.

In our study, no significant association was observed between the tumor stage and Endocan status (*P*=0.31). This finding was in line with the study's findings by Peynirci* et al.* on papillary thyroid carcinoma (27). However, the findings of our study were in contrast to the results of the studies conducted by Irani* et al.* and Kim* et al.* studies (24). 

The lack of association between tumor stage and Endocan status in our study might be related to the low frequency of high stage tumors in this study. This reason may also explain the high expression of Endocan in borderline mucinous tumors, which are more common compared to malignant mucinous tumors. Therefore, there is a need for further studies on a large number of various types of malignant ovarian tumors to identify the validity and reliability of Endocan in assessing malignancy and stage of ovarian tumors. 

## Conclusion

In conclusion, Endocan may be considered as an adjunct in distinguishing malignant ovarian tumors from borderline and benign tumors. 

Moreover, it can be proposed that elevated Endocan-MVD and Endocan expression in tumor vessels are crucial events in cancer formation, tumor differentiation, angiogenesis, and tumor invasion. Furthermore, Endocan may be considered as a tumor marker and a potential new target for cancer therapy. However, further large-scale studies are needed to confirm this proposal.

## Conflict of Interest

The authors declare that there is no conflict of interest regarding the publication of this article.

## Financial Disclosure

The authors declare that there is no financial disclosure.
